# The effects of loving-kindness meditation on doctors’ communication anxiety, trust, calling and defensive medicine practice

**DOI:** 10.1186/s13030-024-00307-7

**Published:** 2024-05-10

**Authors:** Hao Chen, Chao Liu, Kan Wu, Chia-Yih Liu, Wen-Ko Chiou

**Affiliations:** 1 School of Film Television & Communication, Xiamen University of Technology, Xiamen, China; 2 School of Journalism and Communication, Hua Qiao University, Xiamen, 361021 China; 3 Business Analytics Research Center, Chang Gung University, Taoyuan, 33302 Taiwan; 4 Department of Psychiatry, Chang Gung Memorial Hospital, Taoyuan, Taiwan; 5 Department of Industrial Engineering and Management, Ming Chi University of Technology, New Taipei, Taiwan; 6 Department of Industrial Design, Chang Gung University, Taoyuan, Taiwan

**Keywords:** Loving-kindness meditation, Communication anxiety, Trust, Calling, Defensive medicine practice

## Abstract

**Objective:**

The study investigated the effects of loving-kindness meditation (LKM) on doctors’ communication anxiety, trust, calling, and defensive medicine practice.

**Methods:**

This study recruited 94 doctors from a hospital in China, randomized them to an LKM group (*n* = 47), and waited for the control group (*n* = 47). The experimental group accepted an 8-week LKM interference while the waiting for the control group underwent no interference. Researchers measured four major variable factors (communication anxiety, trust, calling, and defensive medicine practice) before and after the LKM intervention.

**Results:**

In the experimental group, trust, and calling were significantly higher, and communication anxiety, and defensive medicine practice were significantly lower than in the control group. In the control group, there were no noticeable differences in any of the four variables between the pre-test and post-test.

**Conclusions:**

The results of this study demonstrate that LKM may help to improve trust, and calling, and reduce communication anxiety and defensive medicine practice. The finding of LKM’s effect extends the understanding of the integrative effects of positive psychology on the decrease of defensive medicine practice.

**Trial registration:**

ChiCTR2300074568. Registered in Chinese Clinical Trial Registry (ChiCTR), 9 August, 2023.

**Supplementary Information:**

The online version contains supplementary material available at 10.1186/s13030-024-00307-7.

## Introduction

Defensive Medicine, a practice wherein medical professionals undertake unnecessary procedures or advise against high-risk procedures to protect themselves from litigation, has emerged as a critical concern in healthcare. Initially identified by Tancredi and colleagues in 1978, this phenomenon not only escalates healthcare costs but also potentially compromises patient care and trust in the medical profession [1]. The act as a diagnostic procedure is not necessary for the medical disease itself, but to construct a complete defense system against possible medical lawsuits. Defensive medicine will bring a lot of negative effects: (1) Patients will experience additional medical risks [2]. (2) Increased costs of diagnosis and treatment and improper use of resources [3]. (3) make the doctor-patient relationship worse [4]. (4) Delay the development of medicine [5].

Given the universality of defensive medicine phenomenon and the harmfulness of defensive medicine behavior, in recent years, the academic circle has discussed the question of why doctors choose defensive medicine. Past studies have found that improving the medical staff’s scientific research skills, establishing risk-coping mechanisms, strengthening medical staff’s professional ethics education, and rebuilding doctor-patient trust can reduce doctors’ defensive medicine [6]. Hospitals should establish and improve the internal responsibility supervision and responsibility-bearing system, organically combine the evaluation results with the deepening labor and personnel distribution system, and effectively reduce doctors’ defensive medical behavior through the combination of system norms and professional ethics constraints [7]. Communication is the basis of rebuilding the doctor-patient trust relationship, effective doctor-patient communication can avoid most unnecessary doctor-patient conflicts, relieve the pressure on doctors, and reduce the occurrence of defensive medical behaviors [8].

Numerous previous studies on defensive medicine were discussed from the perspective of external factors such as litigation pressure, institutional environment, and doctor-patient relationship [9]. Despite extensive discourse on mitigating defensive medicine through legal reform and enhancing doctor-patient communication, an underexplored avenue is the impact of doctors’ psychological well-being on their propensity to engage in such practices. Efforts should be made to balance the doctors’ sense of anxiety and risk perception to prevent defensive medicine [10]. When doctors have stronger emotions about unjustified litigation, they will adopt more defensive medical behaviors [11]. Therefore, the emotional performance of doctors in the face of various external pressures may have an important impact on whether to defensive medical behaviors.

Recent years have seen a growing interest in the application of mindfulness and meditation techniques as interventions to improve various aspects of healthcare delivery [12]. Among these, Loving-Kindness Meditation (LKM) has shown promise in enhancing positive affect, reducing stress, and fostering empathy [13]—qualities that are inversely related to the practice of defensive medicine. However, the direct implications of LKM on defensive medicine practices have received limited attention in empirical research. From the perspective of positive psychology, this study aims to investigate the effectiveness of LKM in mitigating defensive medicine practices among doctors.

### Loving-kindness meditation and defensive medicine practice

LKM, also known as Compassion meditation, promotes positive feelings about oneself and others by sending blessings to real or imagined people, including oneself, benefactors, loved ones, ordinary people, haters, and everyone [14]. It also requires mindfulness and peace while practicing, and helps the practitioner gain a sense of inner cohesion. LKM relieves depression by reducing self-critical thoughts and enhancing feelings of self-compassion. Even short-time LKM for a few minutes can increase feelings of social connection and positive affect toward other individuals on both an explicit and implicit level [15]. LKM refers to a special meditation practice that cultivates compassion, and the object of this compassion is all life, without boundaries, without limits [16]. Kindness is to wish all life happiness, and compassion is to wish all life relief from suffering [17].

Few studies have explored the use of LKM in the workplace, especially its impact on positive affect and defensive medicine. Studies have shown that LKM can improve the positive effect of employees in enterprises [18]. Positive affect falls under the category of pleasure and reflects the joyful tone of subjective experience, so the types of positive affect are those that are enjoyable and cause us to engage in behaviors we enjoy. The emotional experiences contained in positive affect can be more accurately explained and communicated in terms such as confidence, love, sex, passion, attachment, hope, etc [16]. . . Past research has shown a negative correlation between positive affect and defensive medicine among Turkish healthcare workers. While there is evidence that positive affect and defensive medicine are negatively correlated, research exploring the mechanisms behind this relationship is limited [19]. The study of D Montanera [19] simulated interactions between patients, doctors, and health insurers in which doctors can administer both positive and negative defensive medicine. It suggested that this ability to practice two types of defensive medicine leads to the existence of two potential balance types: one in which doctors are willing to accept an equal number of patients with expected illness in a more positive mood (full accessibility), and the other in which doctors are relatively negative in accepting fewer patients (limited accessibility). A’s study found that defensive behavior reflects the underlying emotional state and negative emotions can drive a variety of defensive behaviors. Researchers found that defensive behavior reflects the underlying emotional state and negative emotions can drive a variety of defensive behaviors [20]. Therefore, LKM may have a positive effect on reducing defensive medical behavior, and the mechanism behind it deserves further study.

### Communication anxiety, trust, and calling

Communication anxiety refers to a psychological feeling of tension and anxiety in the face of communication with others or groups [20]. Previous studies have explored the antecedents of communication anxiety and found that positive affect can reduce an individual’s communication anxiety. Positive affect training may reduce defensive medicine by reducing communication anxiety. Some literature also reveals the impact of communication anxiety on defensive medicine. Studies have shown that communication anxiety is positively correlated with the defensive medicine of Indian neurosurgeons [21], Chinese doctors [22], and American doctors [23]. Taken together, these previous findings strongly suggest that people with high levels of positive affect experience fewer levels of communication anxiety, thereby reducing their defensive medicine.

In addition to the fact that communication anxiety may help explain the relationship between positive affect and defensive medicine, trust may play a mediating role between positive affect and defensive medicine. Trust is the assumption of another person’s knowledge, competence, and goodwill that the other person is close to one’s own and will not harm one’s interests [24]. Previous research has explored the antecedents of trust and found that positive affect can promote personal trust. Positive affect training may reduce defensive medicine by enhancing trust. Some literature also reveals the effect of trust on defensive medicine. Studies have shown a negative correlation between trust and defensive medicine among doctors in the UK [25] and oncologists in Italy [26]. Colombian doctors’ trust was negatively correlated with defensive medicine [27], and Israeli doctors’ trust was negatively correlated with defensive medicine [28]Taken together, these previous findings strongly suggest that people with high levels of positive affect experience higher levels of trust, thereby reducing their defensive medicine.

Occupational calling is an individual’s intense passion and strength for a particular field, emphasizing identification and a strong desire for purpose [29]. Previous research has explored the antecedents of a sense of purpose and found that positive affect can promote a sense of purpose. Positive affect is positively correlated with a sense of mission among hotel workers in China [30] and call center workers in the United States [31]. Some literature also reveals the impact of a sense of mission on defensive medicine. Studies have shown a negative correlation between the sense of mission and defensive medicine for obstetricians and gynecologists in the United States [32] and surgeons in Italy [33]. Taken together, these previous findings strongly suggest that people with high levels of positive affect experience higher levels of a sense of purpose, and thus reduce their defensive medicine.

### Research purpose and hypotheses

To sum up, this study aims to explore the mechanism of loving-kindness meditation (LKM) on doctors’ communication anxiety, trust, and defensive medicine. The current research thus seeks to elucidate the role of LKM in fostering a healthcare environment where decisions are driven by patient welfare and evidence-based practice rather than the fear of litigation. Through a rigorous experimental design involving medical professionals, this study examines the transformative potential of LKM in reducing defensive medicine practices, contributing to the dual goals of improving healthcare delivery and ensuring patient safety.

Based on the above literature, theories, and research findings, this study proposes the following hypotheses:

#### Hypothesis 1

(H1): LKM intervention can significantly reduce the level of communication anxiety.

#### Hypothesis 2

(H2): LKM intervention can significantly improve the level of interpersonal trust.

#### Hypothesis 3

(H3): LKM intervention can significantly improve the level of calling.

#### Hypothesis 4

(H4): LKM intervention can significantly reduce the level of defensive medicine practices.

## Methods

### Participants

The participants in this study were doctors working at a hospital in China. The recruitment information was collected through the internal network system of the hospital. In the recruitment information, LKM is a psychosomatic healing activity, which can help release pressure, relax mind and body, relieve anxiety, and cultivate love and empathy. Interested doctors can voluntarily sign up for the study. A total of 94 eligible participants completed the experiment and were randomly assigned to one of two groups: the LKM group (*n* = 47) and the waiting control group (*n* = 47). The demographic information of the participants is shown in Table 1. There was no significant difference in age composition, sex ratio composition, and other factors between the two groups.

**Table 1:** Demographic characteristics of participants

Characteristic		Total (*n* = 94)	LKM Group (*n* = 47)	Control Group (*n* = 47)
Age (SD)		39.27 (9.43)	38.17 (8.88)	40.36 (9.93)
Gender	Male (%)	34 (36.2%)	17 (36.2%)	17 (36.2%)
	Female (%)	60 (63.8%)	30 (63.8%)	30 (63.8%)

### Instruments

#### Communication anxiety inventory (CAI)

This scale was used to measure the subjects’ communication anxiety. Using the CAI developed by Butterfield and Gould, which consists of 7 items, the Likert five-point self-rating scale was adopted. On a scale of 1–5 from “strongly agree” to “strongly disagree” [34]. Past studies have shown quite good validity [35]. The Chinese version of the CAI, adapted by Chen et al., has the same structure [36]. In this study, the Cronbach’s a of this scale was 0.92.

#### Interpersonal trust scale (ITS)

This scale is used to measure subjects’ interpersonal trust. Following Rotter’s ITS, there are 25 items in the scale, and Likert’s five-point self-rating scale is adopted, ranking 1–5 points from “completely agree” to “completely disagree” [37]. Previous studies have shown that it is valid [38]. The Chinese version of the ITS, adapted by Chiou et al., has the same structure [39]. In this study, Cronbach’s a on this scale was 0.91.

#### Brief calling scale (BCS)

This scale was used to measure the subjects’ sense of career mission. BCS compiled by Dik et al., consists of 2 items. Respectively “I have a sense of vocation that drives me to seek a particular type of job” and “I can understand my vocation well because it applies to my vocation”, using a Likert 5-point scale ranging from “1 = safe non-fit” to “5 = perfect fit” [40]. Previous studies have shown quite good validity of this scale [41]. The Chinese version of the BCS, adapted by Liu et al., has the same structure [16]. In this study, the Cronbach’s a of this scale was 0.90.

#### Defensive medicine practices scale (DMPS)

This scale is used to measure the defensive medicine behavior of the subjects. DMPS was developed by Unal and Jung (1995). This scale contains 2 dimensions: active defensive medicine and passive defensive medicine, with a total of 10 questions. The response type is a Likert 5-point scale, “1” means completely inconsistent, and “5” means completely consistent; The total score of each subscale is the score of each subscale, and the sum of 2 subscales is the total scale score [42]. Past studies have shown that it has quite good validity [43]. The Chinese version of the DMPS, adapted by Chiou et al., has the same structure [44]. In this study, the Cronbach’s a of this scale was 0.95.

### LKM intervention

Participants in the LKM group received a 50-minute LKM practical intervention three times a week for a total duration of eight weeks. The interventions were conducted in a group setting, each group consisted of 15–16 participants. The practice location was located in a meditation yoga practice room, which was spacious, quiet, and easily accessible. Due to the doctor’s busy work schedule, the LKM meditation practice is performed every night on weekdays and all day on weekends. The subjects chose to attend three times a week based on their schedule. The meditation practice was conducted by two instructors with more than 3 years of experience in LKM teaching. The instructor did not know the purpose of the study, the specific scheduling of the experiment, and which participants in each activity were involved in the study. Each meditation practice is observed and recorded by researchers who do not interfere with the practice.

Each 50-minute meditation practice consists of the following program: 10 min of coaching, 35 min of LKM practice (with a 5-minute break), and 5 min of exchange experience and discussion. The instructor teaches meditation techniques during a 10-minute guided session before meditation and helps participants relax and unwind into a meditative state. The LKM lasts for 30 min as follows: (1) Adjust to a comfortable sitting position to relax the body and mood; (2) imagine the object being blessed with compassion; Bless the object in four ways: (a) wish that he/she has no enemies, (b) wish that he/she has no suffering, (c) wish that he/she has no disease, and (d) wish that he/she can have his/her happiness. Concerning the choice of the blessed object, the object generally varies according to the level at which the meditator uses kindness meditation, and follows the principle from easy to difficult. The general order is as follows: (a) Bless yourself; (b) bless your loved one; (c) bless those who are neutral, i.e., those whom you neither like nor like; (d) bless those whom you hate; (e) simultaneously and equally bless yourself, your loved ones, the neutral, and the hated; And (f) bless all or all sentient beings. The 5-minute discussion time after the meditation is used for participants to communicate the meditation so that the instructor understands the participants’ meditation experience and answers their questions accordingly. Participants in the LKM group were assigned homework to reinforce the skills learned during the sessions and to encourage daily practice. The homework included daily guided meditation exercises, using audio recordings provided by the instructors, and reflective journaling focusing on experiences and feelings during the meditation practice.

### Procedure

Researchers posted a job advertisement on the internal network of a hospital in China, and doctors who were interested and qualified to participate in the LKM study provided their registration information. The inclusion criteria for subjects were: (1) physicians; (2) more than 2 years of relevant work experience; (3) aged between 25 and 55; (4) Consent to participate in the study and sign an informed consent form. The exclusion criteria are: (1) a history of mental illness or disorder; (b) have taken psychotropic drugs within the last two years, or are currently using psychotropic drugs; (2) have received any form of psychological intervention within the past three years; And (2) experience in any form of meditation training.

Researchers randomly assigned 98 eligible subjects to the experimental group (i.e., the LKM intervention group, 49 subjects) or the control group (i.e., the waiting group, 49 subjects). Grouping personnel used the random sequence code generated by SPSS 23 software to assign groups. These code lists, initially labeled “Group A” and “Group B,” were provided to participants, who were randomly assigned to either Group A or Group B in a 1:1 assignment ratio.

**Fig. 1:**
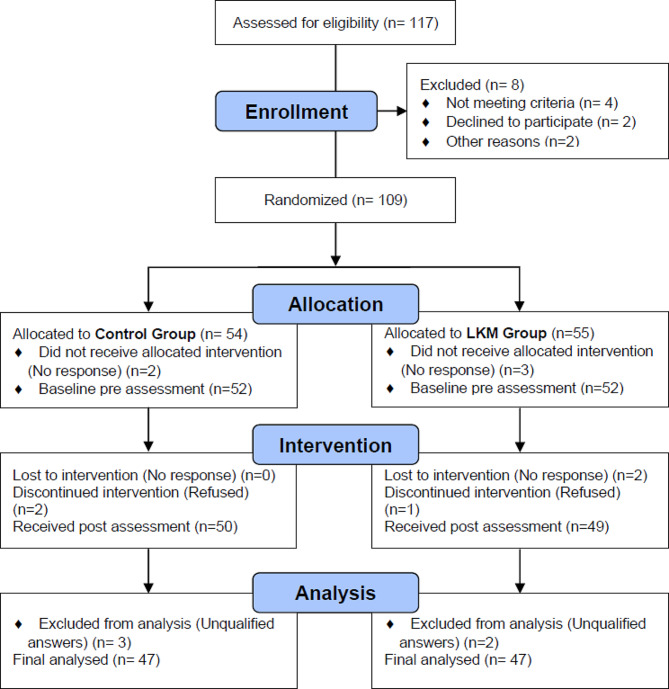
Procedure flow chart

The intervention was conducted by researchers with 10 years of LKM practice experience and two years of meditation teaching experience. The sessions (LKM or wait) were conducted in a quiet, distraction-free room. All participants in the study received 50 Chinese yuan participant fee at the beginning of the test. Each participant was told they were participating in a study related to personality. The participants were then provided with instructions, which the researchers confirmed the participants understood before continuing. After confirming that they understood the instructions, participants provided demographic data and completed the following questionnaires (pretests): (1) the Communication Anxiety Scale (CAI); (2) Interpersonal Trust Scale (ITS); (3) Career Purpose Scale (BCS); And (4) Defensive Medicine Scale (DMP). The time required to answer the questionnaire is approximately 20 min. Participants then completed 30 min of LKM activities. The duration of the LKM intervention was eight weeks. At the end of the intervention period, participants completed the same questionnaire again (after the test) and received another 50 Chinese yuan participant fee. At the end, the participants were explained the real purpose of the study. This study was approved by the Ethics Committee of Changgung University (IRB No. 201902226B0), and the research protocol was carefully reviewed to ensure that it complied with the ethical guidelines of the Chinese Psychological Society. The flow chart of the research procedure is shown in Fig. 1.

Researchers conducted a thorough evaluation of adverse events throughout the study. Participants were asked to report any negative experiences or discomfort they attributed to the intervention during and immediately after the meditation sessions. All reported events were reviewed by the study’s safety monitoring board, consisting of experienced meditators and clinical psychologists, to assess causality and take necessary actions. There have been few previous studies on adverse events caused by LKM exercises. Studies of adverse events associated with mindfulness meditation found that the overall incidence of adverse events was 8.3% [45]. The incidence of adverse events experienced by the subjects in this study was also very low (see Table 2), and the symptoms were mild and resolved after short rest and treatment.

**Table 2:** Adverse events were reported during the LKM intervention

Adverse Event Category	Description	Frequency (person-time)	Severity	Resolved	Actions Taken
Physical Discomfort	Tired	2	Mild	Yes	Sleep
	Increased heart rate	1	Mild	Yes	Deep breath
	Muscle Soreness	3	Mild	Yes	Massage
Psychological &Emotional Discomfort	Anxiety	1	Mild	Yes	Have a rest
	Depression	1	Mild	Yes	Have a rest
Neurological & Cognitive Discomfort	Headache	1	Mild	Yes	Have a rest
	Giddy	2	Mild	Yes	Have a rest
Other	—	0	—	—	—

### Statistical methods

SPSS 23 (IBM, 2015) was used for statistical analysis. The confidence interval was set at 95% and the significance level was set at 0.05. Descriptive statistics were used to describe the basic data distribution of subjects, and Multivariate Analysis of Variance (MANOVA) was used to compare the intervention effects before and after the experiment.

## Results

**Fig. 2:**
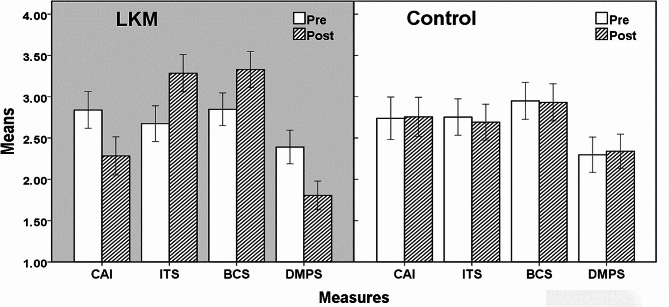
Pairwise comparison between LKM and control group. *Note.* Errors bar: Standard error

This study used a 2(Group: LKM, Control) × 2(Time: Pre, Post) parallel trial design. MANOVA was conducted on communication anxiety (CAI), interpersonal trust (ITS), brief calling (BCS), and defensive medicine practice (DMPS). The descriptive statistics are presented in Table 3; Fig. 2.

**Table 3:** Descriptive statistics

Group	Measures	Mean (SD)	
		Pre	Post
LKM (*n* = 47)	CAI	2.840 (0.759)	2.283 (0.792)
	ITS	2.674 (0.750)	3.284 (0.774)
	BCS	2.849 (0.676)	3.329 (0.741)
	DMPS	2.391 (0.697)	1.806 (0.590)
Control (*n* = 47)	CAI	2.739 (0.885)	2.757 (0.812)
	ITS	2.754 (0.758)	2.692 (0.745)
	BCS	2.950 (0.762)	2.931 (0.769)
	DMPS	2.298 (0.731)	2.341 (0.706)

Note. CAI: Communication Anxiety Inventory; ITS: Interpersonal Trust Scale; BCS: Brief Calling Scale; DMPS: Defensive Medicine Practices Scale

Results showed that there was a significant main effect on the Group: f(6, 179) = 6.257, *p* < 0.001, η^2^ = 0.173; a significant main effect on Time: f(6, 179) = 6.120, *p* < 0.001, η^2^ = 0.214; and a significant interaction effect between Group and Time: f(6, 179) = 11.652, *p* < 0.001, η^2^ = 0.281. Results of Tests of Between-Subjects Effects are shown in Table 4.

**Table 4:** Tests of Between-Subjects Effects

Measures	Variable	F	*p*	η^2^
CAI	Group	2.458	0.119	0.013
	Time^*^	5.147	0.024	0.027
	Group×Time^*^	5.853	0.017	0.031
ITS	Group^*^	5.408	0.021	0.029
	Time^*^	6.188	0.014	0.033
	Group×Time^**^	9.274	0.003	0.048
BCS	Group	1.896	0.170	0.010
	Time^*^	4.604	0.033	0.024
	Group×Time^*^	5.362	0.022	0.028
DMPS	Group^*^	4.906	0.028	0.026
	Time^**^	7.393	0.007	0.039
	Group×Time^**^	9.952	0.002	0.051

Note. CAI: Communication Anxiety Inventory; ITS: Interpersonal Trust Scale; BCS: Brief Calling Scale; DMPS: Defensive Medicine Practices Scale; * *p* < 0.05; ** *p* < 0.01

**Fig. 3:**
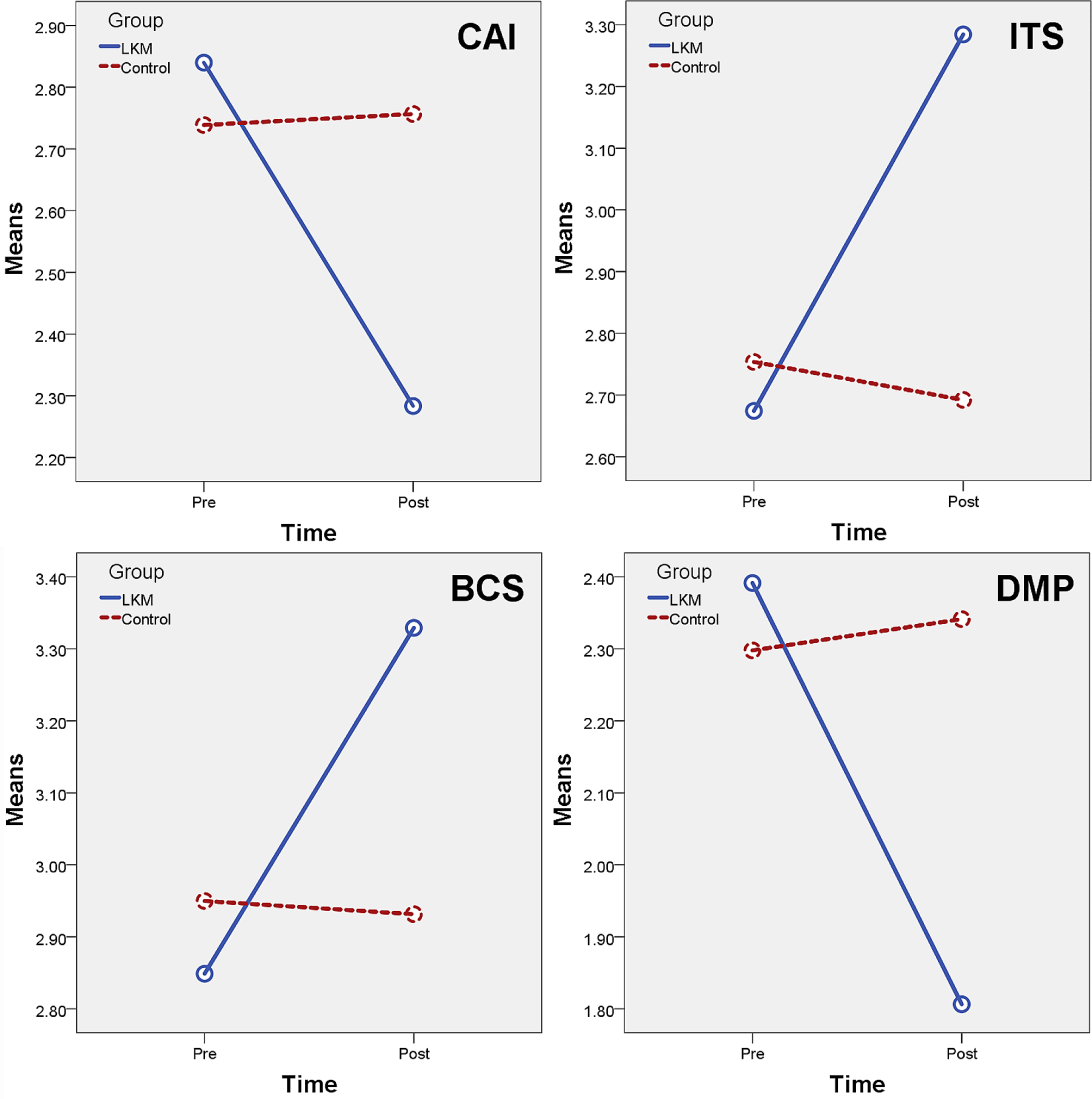
Significant interaction effects between Group and Time

It can be seen from the above results that there were significant Group main effects in all measures except CAI and BCS. All measures have a significant Time main effect and Group × Time interaction effect. These significant interaction effects are shown in more detail in Fig. 3.

The results of this study showed that LKM intervention significantly improved the level of interpersonal trust, and short summoning. It also significantly reduced subjects’ levels of communication anxiety, and defensive medicine practice. So all the hypotheses are supported.

## Discussion

This study investigated the impact of Loving-Kindness Meditation (LKM) on doctors’ communication anxiety, trust, calling, and defensive medicine practices. Consistent with the hypotheses, the findings revealed significant improvements in trust, and calling, alongside reductions in communication anxiety, and defensive medicine practices among participants who engaged in the LKM intervention. These outcomes underscore the potential of LKM as a transformative tool in healthcare settings.

1. LKM can effectively improve attitudes toward oneself and others, and these effects make LKM promising in reducing communication anxiety. Research has found that a kind attitude toward oneself, conceptualized as self-compassion, also helps reduce communication anxiety [46]. The positive effect that results from LKM is expanded focus, which in turn helps to get rid of the negative perceptions and emotions that lead to anxiety about future communication. Studies have shown that LKM effectively reduces communication anxiety under social evaluation pressure [47]. The main reason is that before the introduction of meditation, doctors were more accustomed to wrapping themselves in indifference when facing various strangers [39]. Doctors often have such psychology, that is, fear that they will make mistakes, or a factor of uncertainty, insecurity, and distrust, so that they are always reluctant to express themselves in front of strangers, in the eyes of patients, doctors are generally a pronoun of silence, especially when they see patients together, they are writing medical records with their heads down [36]. After the intervention of meditation, doctors begin to have the courage to open up and release themselves, so they reduce interpersonal communication anxiety [48].
2. LKM can effectively improve individuals’ interpersonal trust. Previous studies have confirmed that emotions have a direct impact on social judgments such as interpersonal trust, which will further affect the development and maintenance of interpersonal trust [49]. Studies have found that positive affect can enhance interpersonal trust, while positive affect can reduce it. Both positive and negative effects help or hinder interpersonal interaction [50]. The increase in positive affect can promote more overlap between oneself and others. Positive affect and interpersonal trust are a virtuous cycle process. The more positive affect an individual has, the higher the level of interpersonal trust, and the stronger the ability to communicate with others [51]. Good interpersonal skills will encourage the individual to produce more positive affect, and then improve the level of interpersonal trust [52]. Previous studies have found that positive qualities can effectively predict involvement in social activities. Positive affect experience has a significant impact on interpersonal trust communication, and there is a significant positive correlation between interpersonal trust and positive affect experience [53]. Positive affect has a very positive impact on individuals’ interpersonal trust, which can make individuals full of energy and more active and positive in interpersonal communication [54]. When the initiative of communication is increased, the individual can establish good interpersonal trust with others. Therefore, LKM can enhance an individual’s interpersonal trust when interacting with others.
3. LKM can effectively enhance an individual’s calling because LKM enhances the meaning of life. The meaning of life refers to the life goal extracted from the life experience of an individual, and it is the experience of an individual who constantly thinks about the relationship between himself and the world [55]. Individuals seeking a sense of purpose are in the process of seeking a meaningful career and expect to build the meaning of life through their careers. The process of seeking a sense of purpose triggered by an individual is the process of seeking meaning [56]. LKM enables individuals to develop from livelihood orientation and career orientation to mission orientation. The bread-oriented person is interested in obtaining material rewards from work and satisfying the necessary resources of life [57]; Career-oriented people think that the purpose of work is more important to obtain promotion, career reputation, and status; Mission-oriented people attach more importance to self-realization in work and the value and contribution of work to society [58]. With the deepening of LKM practice, doctors are never satisfied with their work, full of “burnout” and “complaints”, and gradually turn to pursuing a sense of purpose and meaning in work [59]. They not only regard clinical and scientific research as a “breadwinner” work, or purely pursue status promotion and professional prestige, but also pursue a sense of social public care and the spirit of responsibility. Therefore, LKM can enhance an individual’s sense of mission [60].
4. LKM can effectively reduce the defensive medicine of individuals. The positive effect brought about by LKM often brings a strong self-efficacy to the individual, which prompts them to show greater enthusiasm, thereby exercising competence and forming an advantage [61]. Successful experiences can reflect the knowledge and ability of the individual to complete the task, which are strengths of the individual. Acting based on positive affect and successful experiences in the past are both positive life events experienced by individuals. Identifying strengths through these two ways can help individuals gain positive self-efficacy, better adapt to the environment through strengths, and reduce defensive medicine [36]. In the course of LKM, doctors gradually let their guard down, no longer believe that the current medical environment is intolerant of doctors, the doctor-patient relationship is tense, and the patient is no longer regarded as a potential litigant [48]. Therefore, doctors no longer prescribe various tests and examinations for patients that are not necessary for their actual condition, prepare multiple sets of treatment plans, and actively invite consultations; In addition, in the face of serious patients with greater risks, doctors no longer refuse treatment, but hope to help him out of pain. Therefore, LKM can enhance an individual’s defensive medicine [6].

## Research limitations and prospects

To fully and scientifically validate the hypothesis of this study, five scales and an intervention group design were used for eight consecutive weeks. However, there are some problems in the study, and there are still some issues that need to be improved and further explored: (1) The experimental subjects are only a certain number of doctors, and the universality of the subjects is not enough. From the statistical point of view, the subjects with the original low level have a large room for improvement, and it is easier to conclude that the intervention results are significant. The purpose of this study is to explore the impact of LKM intervention on defensive medicine, and it can have practical effects on all ages and groups. Therefore, it is still necessary to further expand the scope of group intervention to medical staff such as nurses, and whether the research results can be more widely promoted needs further verification. (2) To establish a more standardized, systematic, and actionable LKM intervention program in line with the research requirements, including the targeting of individual emotions, more appropriate and detailed evaluation methods need to be developed. (3) Multiple intervention groups and control groups were added, and the intervention time was extended. Through long-term comparison with the control group, the influence and effect of LKM on various stages of medical workers’ careers were further discussed. (4) The deviation rate between participants’ behavioral intentions and their actual behaviors was observed. Further research could explore the causes of this bias, such as the influence of each factor on the paradox or potential mechanisms induced by binary logit, NCA, fsQCA, or in-depth interviews. Finally, other variables, such as personality traits, mindfulness, safety attitudes, and flow, may also influence defensive medicine intent and behavior. Future research may incorporate these variables to refine the model and improve its explanatory power.

**Declarations**.

### Electronic supplementary material

Below is the link to the electronic supplementary material.

Supplementary Material 1

## Data Availability

The datasets during the current study are not publicly available due to privacy restrictions but are available from the first author on reasonable request.
