# Supplementary material for: The effects of loving-kindness meditation on doctors’ communication anxiety, trust, calling and defensive medicine practice
Source: Biopsychosoc Med. 2024 May 10;18:11. doi: 10.1186/s13030-024-00307-7 (PMC11088149; doi:10.1186/s13030-024-00307-7)
Supplement: Supplementary file 1 — Supplementary Material 1 [file 13030_2024_307_MOESM1_ESM.docx]

We greatly appreciate your thoughtful and constructive review of our manuscript. We are grateful for your recognition of the potential contribution of our research to the field of defensive medicine and for highlighting areas that require clarification or improvement. Below, we address each of your comments to enhance the interpretability and overall quality of our study. The red font in the manuscript is the part we added and modified.

Question 1: I wrote last time that the Introduction was introductory and needed to be revised. In response to that comment, the authors have revised the Introduction, but I still think it is redundant and lacks clarity about the purpose and hypotheses of the study. There are too many hypotheses presented, making it difficult to discern the study's objective. Since the effects of positive and negative emotions in Loving kindness meditation (LKM) have already been verified, it is not necessary to assume their effects in this study. The introduction should be described in accordance with the hypothesis that Loving Kindness Meditation (LKM) is effective in defensive medicine.

Answer 1: In order to improve the clarity and relevance of the introduction, we have removed the redundant content: the widely validated content on the role of positive and negative emotions in loving-kindness meditation has been removed. Clarify research objectives and hypotheses: Focus the introduction on the potential utility of loving-kindness meditation in reducing the practice of defensive medicine. Simplifying hypotheses: Reduce the number of hypotheses and keep only the core hypotheses that are most directly relevant to the research, see lines 123-133 for details.

Question 2: The authors added, " The group list was not obtained during the experimental intervention. After the data analysis was completed, the group staff informed the data analysis staff and the experimental intervention staff about the meaning of the groups in the group list. In this way, double blindness was realized". But I did not understand what the author meant. This study was to examine the effects of the psychotherapy with a waiting group, not to compare its effects with those of placebo.

Answer 2: We have removed the redundant content, see lines 202-206 for details.

Question 3: The authors still stated, "All participants in the study received a gift voucher worth 50 Chinese yuan at the beginning of the test to boost their motivation". I did not understand the authors' explanation either. I suggest that the "to boost their motivation." be deleted and that if it is an honorarium, it be clearly stated.

Answer 3: We have removed the redundant content, see line 210 for details.

Question 4: The authors added, " The correlations of the measured variables are presented in Table 4". However, I could not understand the need for the results of the correlation analyses.

Answer 4: We have removed the Pearson correlation.

Question 5: The discussion should be written from the results of the main objective in this study.

Answer 5: According to your request, we have focused the discussion on the change of four variables, see lines 264-321 for details.

Question 6: As I said, this paper does not seem to follow the guidelines of this journal. For example, PS Kunwar, M Zelikowsky, R Remedios, HJ Cai, M Yilmaz, M Meister and DJ Anderson.

Answer 6: We have modified the reference format according to the requirements of your journal, see lines 92-93 for details.
